# Outcomes of Inhaled Amikacin and Clofazimine-Containing Regimens for Treatment of Refractory *Mycobacterium avium* Complex Pulmonary Disease

**DOI:** 10.3390/jcm9092968

**Published:** 2020-09-14

**Authors:** Bo-Guen Kim, Hojoong Kim, O. Jung Kwon, Hee Jae Huh, Nam Yong Lee, Sun-Young Baek, Insuk Sohn, Byung Woo Jhun

**Affiliations:** 1 Division of Pulmonary and Critical Care Medicine, Department of Medicine, Samsung Medical Center, Sungkyunkwan University School of Medicine, Seoul 06351, Korea; kbg1q2w3e@gmail.com (B.-G.K.); hjk3425@skku.edu (H.K.); ojkwon@skku.edu (O.J.K.); 2 Department of Laboratory Medicine and Genetics, Samsung Medical Center, Sungkyunkwan University School of Medicine, Seoul 06351, Korea; pmhhj77@gmail.com (H.J.H.); micro.lee@samsung.com (N.Y.L.); 3 Statistics and Data Center, Research Institute for Future Medicine, Samsung Medical Center, Seoul 06351, Korea; sun0.baek@gmail.com (S.-Y.B.); insuks@gmail.com (I.S.)

**Keywords:** *Mycobacterium avium*, refractory, inhalation, amikacin, clofazimine

## Abstract

Limited data are available regarding optimal treatment for refractory *Mycobacterium avium* complex-pulmonary disease (MAC-PD). We evaluated outcomes of inhaled amikacin (AMK) with clofazimine (CFZ) regimens as an add-on salvage therapy for refractory MAC-PD. We retrospectively analyzed 52 patients with refractory MAC-PD, characterized by persistently positive sputum cultures despite >6 months of treatment. Thirty-five (67%) patients had *M. intracellulare*-PD, and 17 (33%) patients had *M. avium*-PD. Twenty-seven (52%) patients received the salvage therapy for ≥12 months, whereas 25 (48%) patients were treated for <12 months due to adverse effects or other reasons. Seventeen (33%) patients had culture conversion: 10 (10/27) in the ≥12-month treatment group and seven (7/25) in the <12-month treatment group (*p* = 0.488). Microbiological cure, defined as maintenance of culture negativity, was achieved in 12 (23%) patients; six (6/12) with accompanying symptomatic improvement were considered to have reached cure. Clinical cure, defined as symptomatic improvement with <3 consecutive negative cultures, was achieved in three (6%) patients. Overall, 15 (29%) patients achieved favorable outcomes, including microbiological cure, cure, and clinical cure. Inhaled AMK with CFZ may provide favorable outcomes in some patients with refractory MAC-PD. However, given the adverse effects, more effective strategies are needed to maintain these therapeutic regimens.

## 1. Introduction

Nontuberculous mycobacteria (NTM) are ubiquitous organisms that cause chronic disease, and the burdens of NTM-pulmonary disease (PD) are increasing worldwide [1,2,3]. A guideline of the American Thoracic Society/Infectious Disease Society of America recommended multidrug therapy for *Mycobacterium avium* complex (MAC)-PD consisting of a macrolide (i.e., clarithromycin or azithromycin), ethambutol, and rifamycin (i.e., rifampin or rifabutin) with or without addition of an injectable aminoglycoside [4,5]. However, treatment outcomes are unsatisfactory, and a substantial proportion of patients remain culture-positive with refractory MAC-PD [6,7,8,9]. A recent meta-analysis showed that the overall treatment success rate is as low as 60% [7] and that multidrug regimens are poorly tolerated. Therefore, improved therapeutic options are needed for refractory MAC-PD.

The recent guidelines of the British Thoracic Society and Cystic Fibrosis Foundation/Society recommend regimens that include inhaled amikacin (AMK) or other oral agents [10,11]. In addition, data have recently been reported regarding inhaled AMK with clofazimine (CFZ) for the treatment of refractory NTM-PD [12,13]. Notably, the CONVERT trial showed the benefit of adding liposomal AMK inhalation for refractory MAC-PD [14]. In addition, synergistic effects of inhaled AMK and CFZ against NTM species have been observed in vitro [15]. Nevertheless, limited data are available regarding the efficacy of regimens that include inhaled AMK and CFZ in refractory MAC-PD. Therefore, we evaluated the treatment outcomes of inhaled AMK and CFZ regimens as salvage therapy for refractory MAC-PD.

## 2. Materials and Methods

### 2.1. Study Patients

**Figure 1:**
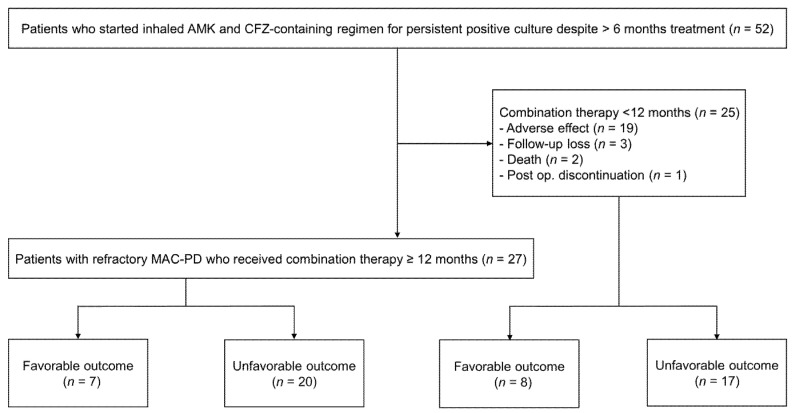
Flow diagram of study population.

We retrospectively analyzed 52 patients with refractory MAC-PD, defined as having persistently positive cultures after >6 months of standardized treatment, who received a regimen containing inhaled AMK with CFZ from January 2016 to June 2018 at the Samsung Medical Center (clinicaltrials.gov identifier NCT00970801) (Figure 1). All patients met the diagnostic criteria for MAC-PD [4]. Data from five of these 52 patients were published previously in another report [12].

This study was approved by the Institutional Review Board (approval no. 2019-07-121). Data were collected with informed consent from patients included in the prospective study, and data on the patients included in this study were analyzed retrospectively. Follow-up data were last updated on 10 July 2019.

### 2.2. Inhaled AMK and CFZ Therapy

Commercially available intravenous AMK sulfate solution (250 mg/mL) was used for inhalation, via a model NE-C28 compressor nebulizer (Omron Colin Co., Ltd., Tokyo, Japan). Before September 2016, patients were treated with 500 mg of inhaled AMK once daily. However, several ototoxicity events occurred, and thus the protocol was changed from 500 mg once daily to 500 mg three times weekly [12]. Oral CFZ was initially prescribed at 100 mg once daily.

Patients underwent laboratory and audiometry examinations at approximately 3-month intervals at an outpatient clinic. Nephrotoxicity was defined as an increase in serum creatinine of ≥0.5 mg/dL from baseline [16]. Ototoxicity was defined as hearing loss of ≥10 dB at two or more consecutive frequencies, hearing loss of ≥20 dB at one isolated frequency, or loss of a response at any three consecutive frequencies on a posttreatment audiogram, in either ear at any frequency [16].

### 2.3. Assessment of Symptomatic, Radiological, and Microbiological Response

Symptomatic responses were assessed by the chronic obstructive pulmonary disease assessment test (CAT) score as improved, unchanged, or worsened, based on a change in CAT score of ≥2 points from baseline [17]. A chest radiograph or computed tomography (CT) image was performed at the initiation of therapy, as well as during follow-up. The radiological form was categorized as fibrocavitary (FC) or nodular bronchiectatic (NB) [18]. According to the presence of a cavitary lesion, NB was additionally classified as a cavitary or non-cavitary form.

Sputum acid-fast bacillus (AFB) smears and cultures were performed using standard methods at 1–3 months intervals [19]. Specimens were cultured both on 3% Ogawa solid medium (Shinyang, Seoul, Korea) and in liquid broth medium in mycobacterial growth indicator tubes (MGIT; Becton, Dickinson and Co., Sparks, MD, USA). For semi-quantitative culture analysis, each culture was scored using methods described in previous studies [20,21]. NTM species identification was conducted via nested multiplex polymerase chain reaction and a reverse-hybridization assay of the internal transcribed spacer region (AdvanSureTM Mycobacteria GenoBlot Assay; LG Life Sciences, Seoul, Korea). Drug susceptibility testing was performed using the broth microdilution method. MAC isolates were evaluated in terms of their resistance to clarithromycin (minimum inhibitory concentration (MIC ≥ 32 μg/mL) and to AMK (MIC ≥ 64 μg/mL) [22].

### 2.4. Evaluation of Treatment Outcomes

Treatment outcomes were assessed at the end of the inhaled AMK and CFZ therapy as follows [23]. “Culture conversion” was defined as at least three consecutive negative sputum cultures after treatment, collected at least four weeks apart. The time of conversion was defined as the date of the first negative culture. “Microbiological cure” was defined as no positive cultures of causative species after culture conversion until the time of outcome measurement. “Clinical cure” was defined as improved symptoms during treatment without available culture to prove culture conversion or microbiological cure (<3 consecutive negative cultures). “Cure” was defined as fulfillment of both clinical and microbiological cure. In this study, microbiological cure, cure, and clinical cure were classified as favorable outcomes. For analysis, we categorized the patients according to the treatment duration of 12 months based on recommended minimum treatment period [5].

### 2.5. Statistical Analysis

Data were presented as numbers (percentages) or medians (interquartile ranges (IQR)). Data were compared by the Mann-Whitney U test for continuous variables and the chi-squared test or Fisher’s exact test for categorical variables. We analyzed the trend of serial changes of semi-quantitative sputum culture positivity by generalized estimating equations. Logistic regression analysis with backward stepwise selection (with *p* < 0.05 for entry and *p* > 0.10 for removal of variables) was used to identify independent factors associated with a favorable outcome. Differences were considered statistically significant at *p* < 0.05. All statistical analyses were performed using SPSS (IBM SPSS Statistics ver. 25, Chicago, IL, USA).

## 3. Results

### 3.1. Baseline Characteristics

Among the 52 patients, the median age was 59 (IQR, 51–70) years, and 48% of them were women (Table 1). More than half (56%) of the patients were never-smokers. The most common underlying condition was previous, treated, pulmonary tuberculosis (52%), followed by chronic pulmonary aspergillosis (33%). Approximately two-thirds (67%) of the patients had *M. intracellulare*-PD, while one-third (33%) had *M. avium*-PD. Twenty-seven patients (52%) had the NB form of MAC-PD with (10/27) or without (17/27) a cavity, while the remaining had the FC form. Positive sputum AFB smears were identified in 34 (65%) patients. Macrolide resistance was identified in 25 (48%) patients, although none had pre-treatment isolates resistant to AMK (Table 1).

### 3.2. Antibiotic Treatment Regimens

All patients had persistent positive cultures after previous antibiotic therapy, which was received for a median treatment duration of 28.5 (IQR, 20.3–55.5) months. Before initiation of the inhaled AMK with CFZ therapy, all patients received azithromycin and ethambutol-based regimens that included rifamycin (96%) or moxifloxacin (25%) (Table 2). Thirty (58%) patients had previously received injectable aminoglycoside for a median of 7.0 (IQR, 5.5–12.0) months.

Regarding the dosage of inhaled AMK, 35 (67%) patients began treatment with 500 mg three times weekly; nine (17%) patients changed from 500 mg once daily to three times weekly because of a protocol change in September 2016 in our institution; and 8 (16%) patients began treatment with 500 mg once daily. All patients received 100 mg of oral CFZ at the initiation of treatment. After initiation of the inhaled AMK and CFZ therapy, 27 (52%) patients maintained the regimen for ≥12 months, while 25 (48%) patients discontinued the regimen in less than 12 months (Figure 1). The median duration of AMK and CFZ therapy in all study patients was 11.9 (IQR, 4.7–18.8) months. Companion drugs, including azithromycin (98%), ethambutol (96%), rifamycin (21%), moxifloxacin (19%), and linezolid (2%), were used in combination with inhaled AMK and CFZ during the study period (Table 2). At the time of starting AMK and CFZ combination therapy, we usually used CFZ instead of rifamycin due to concerns about hepatotoxicity caused by concomitant use of CFZ and rifamycin. In cases with development of macrolide resistance, azithromycin was maintained for its anti-inflammatory effect in our institution.

### 3.3. Treatment Response of Study Patients

Treatment responses were evaluated at the end of the inhaled AMK and CFZ therapy (Table 3). Of the 52 patients, symptomatic and radiological improvement were observed in 25 (48%) and 17 (33%), respectively. Twenty-two (42%) patients had at least one negative culture; sputum culture conversion was achieved by 17 (33%) patients, although nine (53%) of these 17 patients had clarithromycin-resistant isolates. Microbiological cure was achieved in 12 (23%) patients, including five (5/12, 42%) patients with clarithromycin-resistant isolates, but only six (6/12) of them had accompanying symptomatic improvement and were thus considered to have achieved cure. Three (6%) patients who showed improvement of symptoms with <3 consecutive negative cultures were considered to exhibit clinical cure; one (33%) of the three had clarithromycin-resistant isolates. Overall, 15 (29%) patients achieved a favorable outcome, although 47% (7/15) of these patients had clarithromycin-resistant isolates. Sputum culture conversion was achieved after a median of 3.0 months of treatment, and microbiological cure was achieved after a median of 1.1 months of treatment (Supplementary Figure S1A,B). In addition, among the 25 patients who initially had clarithromycin-resistant isolates, culture conversion, microbiological cure, and favorable outcome were achieved in 36% (9/25), 20% (5/25), and 28% (7/25), respectively.

According to the culture results on solid medium, 46 (46/52, 88%) patients had ≥1+ AFB culture positivity at the time of initiation of therapy. Thirteen (13/27, 48%) patients had ≥1+ AFB culture positivity after 12 months of inhaled AMK and CFZ therapy. There was a significant reduction in semiquantitative sputum culture positivity after treatment (*p* < 0.001). In addition, among the 35 patients who did not achieve culture conversion during the study period, semiquantitative culture positivity also tended to decrease significantly (*p* = 0.012) (Table S1).

For semi-quantitative culture analysis, each culture was scored as follows: (i) negative, no growth in liquid or solid medium; (ii) liquid only, growth in liquid medium only; (iii) trace, growth of <50 colonies on solid medium; (iv) 1+, growth of 50 to 100 colonies on solid medium; (v) 2+, growth of 100 to 200 colonies on solid medium; (vi) 3+, growth of 200 to 500 colonies on solid medium; and (vii) 4+, growth of >500 colonies on solid medium.

### 3.4. Adverse Effects Associated with Inhaled AMK and CFZ

During the study period, 24 (46%) and 14 (27%) patients experienced adverse effects associated with inhaled AMK and CFZ, respectively (Table 4). Seventeen patients discontinued inhaled AMK after a median of 3.0 (IQR, 1.1–6.6) months, and seven patients had a change in inhaled AMK dosage: 500 mg once daily to 500 mg three times weekly (*n* = 4), 500 mg three times weekly to 500 mg twice weekly (*n* = 2), and 500 mg three times weekly to 250 mg three times weekly (*n* = 1).

Four (8%) patients discontinued CFZ after 1.7 (IQR, 1.2–5.2) months, and ten (19%) patients had dosages of CFZ changed from 100 mg once daily to 50 mg once daily.

### 3.5. Factors Associated with a Favorable Outcome

Factors associated with a favorable outcome of refractory MAC-PD were explored (Table 5). In multivariable analysis, low ESR was significantly associated with a favorable outcome in all patients (adjusted odds ratio, 0.950; 95% confidence interval, 0.914–0.988).

## 4. Discussion

In this study, more than one-quarter (29%) of patients with refractory MAC-PD achieved a favorable outcome after initiation of inhaled AMK- and CFZ-containing regimens. Approximately one-third (33%) of patients had culture conversion, and microbiological cure occurred in 23% of patients. Our data suggest that regimens containing inhaled AMK with CFZ may yield favorable outcomes in some patients with refractory MAC-PD.

One of the most notable findings in our study was the high proportion of patients with strains that had pretreatment drug resistance. Approximately half (48%) of our patients had clarithromycin-resistant MAC strains at the initiation of the inhaled AMK and CFZ therapy. In previous studies of refractory MAC-PD, which showed culture conversion rates similar to our study, the proportions of patients with macrolide-resistant MAC strains were not high. In a study that included 23 patients with refractory MAC-PD who were treated with inhaled AMK therapy, 39% of patients had clarithromycin-resistant strains prior to treatment, and the conversion rate was 43% [24]. In a recent CONVERT study that evaluated the efficacy of AMK liposome inhalation suspension for patients with refractory MAC-PD, culture conversion was achieved by 29% of patients; however, only 22.9% of patients had pretreatment clarithromycin resistance [14]. Macrolides are the cornerstone of MAC-PD therapy, and development of macrolide resistance, mainly due to point mutations in the 23S rRNA gene, is associated with poor treatment outcomes [25,26,27,28,29]. Thus, given that 36% (9/25) of our patients with clarithromycin-resistant strains experienced culture conversion, inhaled AMK- and CFZ-containing regimens may be worth consideration as treatment strategies, especially for patients with refractory MAC-PD.

AMK is one of the most important antibiotics for treatment of MAC-PD. Only 6.8–10.4% of clinical strains of MAC are reportedly resistant to AMK [30], and a recent study indicated that use of aminoglycosides for ≥3 months was associated with treatment success in cavitary MAC-PD [31]. Inhaled AMK has been used to reduce adverse effects associated with long-term use of injectable AMK and to increase the therapeutic effect through lung absorption [32]. However, studies regarding the efficacy of inhaled AMK for refractory NTM-PD have shown varying culture conversion rates of 18–67% [12,24,32,33]. These discrepancies may have been related to various epidemiological or clinical factors in the study cohorts. Notably, our patients had a long previous median treatment period of 28.5 months; 67% (35/52) of patients had cavitary lesions, which are known to be associated with poor response [18], and this high percentage of cavitary disease cases potentially contributed to the less-than-satisfactory treatment outcomes of many of our patients. Unfortunately, limited data are available regarding whether the differences in effectiveness of inhaled AMK depend on certain patient characteristics, such as the presence of cavitary lesions. Therefore, further studies are needed regarding the efficacy of inhaled AMK for refractory MAC-PD.

CFZ has a number of advantages for treatment of NTM-PD, including its long half-life, slow metabolic elimination, ability to achieve high concentrations in macrophages, and rapid localization within phagocytes. Laboratory studies have demonstrated synergistic effects of CFZ and AMK against NTM [15], and CFZ has been reported to be effective in treatment of MAC-PD [13,34,35]. However, unlike the laboratory findings, these clinical studies yielded inconsistent results. Moreover, appropriate criteria have not been established regarding an optimal MIC threshold of CFZ for MAC-PD, and further research is warranted on this issue.

In our study, approximately half (48%) of the patients discontinued regimens containing inhaled AMK and CFZ within 12 months, mostly due to adverse effects. Eventually, 33% (17/52) of patients discontinued the inhaled AMK, and 8% (4/52) of patients discontinued CFZ. The discontinuation rate of inhaled AMK tended to be higher in our study than in previous reports [12,24]. In a previous study, 27% (21/77) of patients with refractory NTM-PD discontinued inhaled AMK [12], and only 8% (2/26) of patients with refractory MAC-PD discontinued inhaled AMK in a Japanese study, despite the use of similar doses of AMK [24]. These differences were presumably because our patients had a high rate of previous usage of injectable aminoglycoside (58%) and long periods of previous aminoglycoside use (median, 7.0 months). Generally, CFZ is considered tolerable with no severe adverse effects. In our study, the rate of drug discontinuation due to CFZ side effects was not high, with only a few patients having troublesome adverse effects.

Notably, in our study, treatment for more than 12 months was not significantly associated with favorable outcome in multivariable analysis. This may have been due to the small numbers of patients included in the study. However, our data showed that negative culture conversion was achieved in some patients with treatment for less than 12 months. Further studies are required to confirm this point.

This study has several limitations. First, our data may not be generalizable to other geographic areas and clinical settings. Second, changes in symptoms were assessed using CAT scores, but there is little evidence that it is reasonable to use CAT scores to assess symptom changes in patients with NTM-PD. Third, treatment outcomes were reported based on combination therapy with a relatively short duration. Fourth, a recent study discussed the importance of the CFZ MIC level, but we did not measure the MIC levels of CFZ in this study [36]. Fifth, we included “clinical cure” in the definition of “favorable outcome” because patients who achieved “clinical cure” showed clinical and radiological improvement, and sputum samples could not be obtained in most cases with “clinical cure” due to decreased volume of sputum. However, it is possible that our data overestimated the efficacy of the treatment regimens. Finally, because 15 patients had NTM strains with intermediate resistance to AMK (MIC 32 μg/mL), the efficacy of our regimen containing inhaled AMK and CFZ may seem weak.

In conclusion, this is the first report of patients with refractory MAC-PD who were treated with inhaled AMK- and CFZ-containing regimens in a standard clinical setting. Our data showed that these regimens could provide favorable outcomes for some patients. However, given the adverse effects, a more effective strategy is needed to maintain these therapeutic regimens.

## Figures and Tables

**Table 1:** Characteristics of the 52 patients at initiation of inhaled AMK with CFZ regimens.

Characteristics	Value
Age, years	59 (51–70)
Female	25 (48%)
Body mass index, kg/m^2^	20.7 (18.3–22.2)
Weight, kg	54 (48–62)
Never-smoker	29 (56%)
Underlying condition	
Previous pulmonary tuberculosis	27 (52%)
Chronic pulmonary aspergillosis	17 (33%)
Chronic obstructive pulmonary disease	14 (27%)
Previous lung cancer *	3 (6%)
Diabetes mellitus	4 (8%)
Chronic liver disease	2 (4%)
Chronic kidney disease	1 (2%)
Rheumatoid disease	1 (2%)
Other malignancy ^†^	2 (4%)
Etiologic organism	
*M. intracellulare*	35 (67%)
*M. avium*	17 (33%)
Radiologic findings	
Nodular bronchiectatic form	27 (52%)
With cavity	10/27 (37%)
Without cavity	17/27 (63%)
Fibrocavitary form	25 (48%)
Laboratory findings	
Sputum AFB smear positivity	34 (65%)
Serum albumin, g/dL	4.1 (3.8–4.4)
C-reactive protein, mg/dL	0.6 (0.1–1.7)
Erythrocyte sedimentation rate, mm/h	30.0 (18.0–67.3)
FEV_1_, %	68 (53–81)
FVC, %	74 (56–90)
Macrolide resistance, ≥32 μ/mL	25 (48%)
MIC level of AMK	16 (16–32)
4–16 μg/mL	37 (71%)
32 μg/mL	15 (29%)

Data are presented as *n* (%) or median (interquartile range). AMK, amikacin; CFZ, clofazimine; AFB, acid-fast bacilli; FEV_1_, forced expiratory volume-one second; FVC, forced vital capacity; MIC, minimum inhibitory concentration. * No patients had active cancer after lobectomy (*n* = 2) or chemoradiotherapy (*n* = 1). ^†^ Pancreatic cancer (*n* = 1) and thymoma (*n* = 1).

**Table 2:** Antibiotic treatment regimens.

Variables	*N* = 52	Treatment Duration (Months)
Drugs before starting inhaled AMK and CFZ therapy		
Azithromycin	52 (100%)	27.0 (19.0–56.0)
Ethambutol	52 (100%)	27.0 (16.0–56.0)
Rifamycin	50 (96%)	28.0 (19.0–56.0)
Moxifloxacin	13 (25%)	12.0 (6.0–16.0)
Aminoglycoside injection	30 (58%)	7.0 (5.5–12.0)
AMK	2 (4%)	3.0, 8.0
Streptomycin	28 (54%)	7.0 (6.0–12.0)
Total duration before starting AMK and CFZ therapy	52 (100%)	28.5 (20.3–55.5)
Inhaled AMK and CFZ therapy		
Total duration of inhaled AMK and CFZ	52 (100%)	11.9 (4.7–18.8)
≥12 months	27/52 (52%)	18.7 (12.6–26.4)
<12 months	25/52 (48%)	4.6 (1.2–6.6)
Total duration of inhaled AMK	52 (100%)	12.9 (7.2–20.6)
Total duration of CFZ	52 (100%)	12.5 (5.5–18.9)
Companion drugs used with inhaled AMK and CFZ		
Azithromycin	51 (98%)	15.4 (12.4–20.9)
Ethambutol	50 (96%)	16.3 (12.7–22.2)
Rifamycin	11 (21%)	4.0 (3.0–13.0)
Moxifloxacin	10 (19%)	8.5 (3.0–14.2)
Linezolid	1 (2%)	12.4
Total duration after starting AMK and CFZ therapy	52 (100%)	15.4 (12.5–21.2)

Data are presented as *n* (%) or median (interquartile range). AMK, amikacin; CFZ, clofazimine.

**Table 3:** Treatment response at the end of the inhaled AMK- and CFZ-containing regimens.

Variables	*N* = 52
Symptomatic response by CAT score change	
Improved	25 (48%)
Unchanged	11 (21%)
Worsened	16 (31%)
Radiological response	
Improved	17 (33%)
Unchanged	20 (39%)
Worsened	15 (29%)
Microbiological response	
At least one sputum negative culture	22 (42%)
Time to at least one culture negative, months	5.1 (1.0–10.1)
Culture conversion	17 (33%)
Time to culture conversion, months	3.0 (1.0–9.2)
Microbiological cure	12 (23%)
Cure	6 (12%)
Clinical cure	3 (6%)
Favorable outcome	15 (29%)
Microbiological response in ≥12 months treatment group (*n* = 27)	
Culture conversion	10/27 (37%)
Time to culture conversion, months	3.0 (0.9–9.3)
Microbiological cure	7/27 (26%)
Cure	2/27 (7%)
Clinical cure	0/27 (0%)
Microbiological response in <12 months treatment group (*n* = 25)	
Culture conversion	7/25 (28%)
Time to culture conversion, months	3.0 (1.0–9.7)
Microbiological cure	5/25 (20%)
Cure	4/25 (16%)
Clinical cure	3/25 (12%)
Death *	6 (12%)
Time from starting inhaled AMK and CFZ to death, months	11.1 (5.8–20.5)

Data are presented as *n* (%) or median (interquartile range). AMK, amikacin; CFZ, clofazimine; CAT, chronic obstructive pulmonary disease assessment test. * Two patients died before completion of 12 months treatment of AMK and CFZ therapy due to progression of MAC-PD. The remaining four patients died of pneumonia (*n* = 2), pancreatic cancer (*n* = 1), and unknown cause (*n* = 1).

**Table 4:** Adverse effects associated with inhaled AMK and CFZ therapy in 52 study patients.

	Discontinuation	Dose Change	Total
Amikacin inhalation			
Ototoxicity	12 (23%)	5 (10%)	17 (33%)
Fatigue	2 (4%)	0 (0%)	2 (4%)
Tinnitus	1 (2%)	0 (0%)	1 (2%)
Dizziness	1 (2%)	0 (0%)	1 (2%)
Nausea	1 (2%)	0 (0%)	1 (2%)
Hoarseness	0 (0%)	1 (2%)	1 (2%)
Nephrotoxicity	0 (0%)	1 (2%)	1 (2%)
Total	17 (33%) *	7 (14%) ^†^	24 (46%)
Clofazimine			
Skin color change	1 (2%)	9 (17%)	10 (19%)
Loss of appetite	1 (2%)	0 (0%)	2 (4%)
Diarrhea	0 (0%)	1 (2%)	1 (2%)
Fatigue	1 (2%)	0 (0%)	1 (2%)
Hepatotoxicity (>3 times the normal level)	1 (2%)	0 (0%)	1 (2%)
Total	4 (8%) ^‡^	10 (19%)	14 (27%)

Data are presented as *n* (%). AMK, amikacin; CFZ, clofazimine. * Median time from starting inhaled AMK to discontinuation was 3.0 months. ^†^ AMK dosage changes: 500 mg once daily to 500 mg three times weekly (*n* = 4), 500 mg three times weekly to 500 mg two times weekly (*n* = 2), and 500 mg three times weekly to 250 mg three times weekly (*n* = 1). ^‡^ Median time from starting CFZ to discontinuation was 1.7 months.

**Table 5:** Univariate and multivariable analyses of factors associated with favorable outcome (*N* = 52).

Variable	Favorable Outcome * (*n* = 15)	Univariable		Multivariable	
		Unadjusted OR (95% CI)	*p* Value	Adjusted OR (95% CI)	*p* Value
Age ≤ 65 years	2 (13%)	4.432 (0.871–22.550)	0.073		
Female	9 (60%)	1.969 (0.581–6.673)	0.277		
Body mass index, kg/m^2^		1.058 (0.857–1.308)	0.599		
Never-smoker	10 (67%)	1.895 (0.542–6.628)	0.317		
No previous pulmonary tuberculosis	8 (53%)	1.345 (0.404–4.477)	0.630		
No chronic obstructive pulmonary disease	14 (93%)	7.583 (0.894–64.331)	0.063		
*M. avium* (reference: *M. intracellulare*)	8 (53%)	3.556 (1.006–12.562)	0.049		
Negative sputum AFB smear	6 (40%)	1.389 (0.401–4.806)	0.604		
No cavity	7 (47%)	2.362 (0.679–8.222)	0.177		
No macrolide resistance	8 (53%)	1.083 (0.325–3.602)	0.897		
Amikacin MIC < 32 μg/mL	11 (73%)	1.163 (0.303–4.461)	0.825		
Treatment duration ≥12 months	10 (67%)	2.353 (0.672–8.239)	0.181		
FEV_1_ > 60%	14 (93%)	9.545 (1.132–80.506)	0.038		
ESR, mm/h ^†^		0.950 (0.914–0.988)	0.010	0.950 (0.914–0.988)	0.010
CRP, mg/dL ^†^		0.228 (0.057–0.904)	0.035		

OR, odds ratio; CI, confidence interval; AFB, acid-fast bacilli; MIC, minimum inhibitory concentration; FEV_1_, forced expiratory volume-one second; ESR, erythrocyte sedimentation rate; CRP, C-reactive protein. * favorable outcome included microbiological cure, cure, and clinical cure. ^†^ ESR and CRP were measured at the time of starting combination therapy.

## Supplementary Materials

The following are available online at https://www.mdpi.com/2077-0383/9/9/2968/s1, Figure S1: Cumulative (A) culture conversion rate and (B) microbiological cure rate among patients achieving these outcomes, Table S1: Semiquantitative sputum cultures of patients who did not achieve culture conversion on inhaled amikacin- and clofazimine-containing regimens.

## Abbreviations

NTM: nontuberculous mycobacteria; PD, pulmonary disease; MAC, *Mycobacterium avium* complex; AMK, amikacin; CFZ, clofazimine; MIC, minimum inhibitory concentration; CAT, chronic obstructive pulmonary disease assessment test; CT, computed tomography; FC, fibrocavitary; NB, nodular bronchiectatic; AFB, acid-fast bacillus; MGIT, mycobacterial growth indicator tubes; IQR, interquartile range; ESR, erythrocyte sedimentation rate; FEV_1_, forced expiratory volume in 1 s.
