# Supplementary material for: Outcomes of Inhaled Amikacin and Clofazimine-Containing Regimens for Treatment of Refractory Mycobacterium avium Complex Pulmonary Disease
Source: J Clin Med. 2020 Sep 14;9(9):2968. doi: 10.3390/jcm9092968 (PMC7565500; doi:10.3390/jcm9092968)
Supplement: Supplementary file 1 [file jcm-09-02968-s001.pdf]

Table 1. Semiquantitative sputum cultures of patients who did not achieve culture conversion on inhaled amikacin- and clofazimine-containing regimens ($N = 35$).

Patients No.	Baseline (At the Start of Therapy)	3 Months	6 Months	9 Months	12 Months
1 *M. avium*	Trace	Trace	Trace	Negative	Trace
2 *M. avium*	Trace	Trace	Trace	Trace	Trace
3 *M. avium*	1+	1+	Trace	Trace	Trace
4 *M. avium*	2+	2+	2+	Trace	
5 *M. avium*	Trace	*	Trace		Trace
6 *M. avium*	Trace	Trace	1+	Trace	Trace
7 *M. avium*	Trace	Trace	Trace	Trace	Negative
8 *M. avium*	Trace	Negative	Liquid only	Liquid only	Negative
9 *M. avium*	Trace	4+	3+	2+	1+
10 *M. avium*	1+	Trace			
11 *M. intracellulare*	Trace	Liquid only			
12 *M. intracellulare*	3+				
13 *M. intracellulare*	Trace	Liquid only	.	Liquid only	Trace
14 *M. intracellulare*	1+	1+	1+	1+	
15 *M. intracellulare*	2+	Trace	Liquid only	Liquid only	Liquid only
16 *M. intracellulare*	1+	Trace			
17 *M. intracellulare*	1+	1+	Trace	Liquid only	Liquid only
18 *M. intracellulare*	Trace	Trace	3+	Trace	Trace
19 *M. intracellulare*	1+	2+			
20 *M. intracellulare*	Trace	2+	Negative	Liquid only	Liquid only
21 *M. intracellulare*	1+	1+	Trace	Liquid only	
22 *M. intracellulare*	2+	2+	Trace	Trace	
23 *M. intracellulare*	1+	1+	1+		
24 *M. intracellulare*	Liquid only				
25 *M. intracellulare*	1+	1+	Trace		
26 *M. intracellulare*	1+	1+	1+	.	2+
27 *M. intracellulare*	Liquid only	Trace	Trace	2+	1+
28 *M. intracellulare*	Trace	1+			
29 *M. intracellulare*	1+	Trace			
30 *M. intracellulare*	Trace	Trace	1+	Trace	
31 *M. intracellulare*	1+	1+			
32 *M. intracellulare*	Trace	1+	Trace	Trace	Trace
33 *M. intracellulare*	1+	Trace			
34 *M. intracellulare*	Trace	Trace	Trace	Trace	1+
35 *M. intracellulare*	Trace	1+	Trace		

* Patient's sputum was not collected at that time.

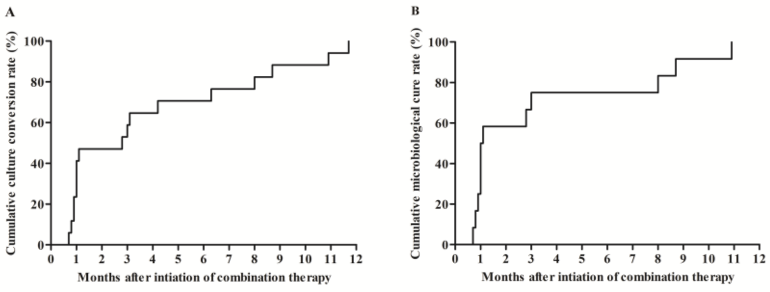

Figure S1. Cumulative (**A**) culture conversion rate and (**B**) microbiological cure rate among patients achieving these outcomes.
